# Vascular endothelial growth factor upregulates expression of annexin A2 in vitro and in a mouse model of ischemic retinopathy

**Published:** 2009-06-17

**Authors:** ShiHong Zhao, LiNa Huang, JinHui Wu, Yuan Zhang, DongYan Pan, Xin Liu

**Affiliations:** Department of Ophthalmology, Changhai Hospital, Second Military Medical University, Shanghai, Peoples Republic of China

## Abstract

**Purpose:**

Annexin A2 has been shown to play a role in many neovascularization diseases. We investigated the effect of vascular endothelial growth factor (VEGF) on annexin A2 expression and related intracellular signaling mechanisms in a mouse model of ischemia-induced retinal neovascularization.

**Methods:**

Annexin A2 expression and the effect of vascular endothelial growth factor (VEGF) on annexin A2 ex pression in retinal neovascularization were assayed by real-time PCR, western blot analysis. The effect of Annexin A2 on retinal neovascularization were assayed by siRNA interference and overexpression of Annexin A2, fluorescence imaging, and immunofluorescence histochemistry in a mouse model of ischemia-induced retinal neovascularization.

**Results:**

Expression of annexin A2 mRNA and protein were increased in the mouse model of ischemia-induced retinal neovascularization and in RF/6A cells treated with VEGF. In RF/6A cells, increased expression of annexin A2 was inhibited by the VEGF receptor 2 (VEGFR2) inhibitor SU10944, and by anti-VEGFR2 neutralizing antibody, and was increased by VEGF. Mice with ischemic retinopathy showed increased expression of annexin A2 in vascular endothelial cells, and enhanced retinal neovascularization after intraocular injection of an adenoviral vector containing an annexin A2 expression cassette. Conversely, annexin A2 knockdown suppressed retinal neovascularization in these mice.

**Conclusions:**

These findings suggest that annexin A2 might induce retinal neovascularization through a VEGF-VEGFR2 pathway in ischemia-induced retina neovascularization. Therefore, annexin A2 is an angiogenesis activator and may be a potential target for the development of effective therapeutic strategies for the treatment of retinal neovascularization.

## Introduction

Retinopathy is a major complication of diabetes mellitus and one of the leading causes of vision loss. Studies have revealed that vascular endothelial growth factor (VEGF) is an important element for many angiogenic processes such as diabetic retinopathy and tumor neovascularization [1,2]. Thus, there is heightened interest in understanding the importance of annexin A2 in regulating the retinal angiogenic process.

The targets of VEGF are two homologous but distinct tyrosine kinase receptors: the feline McDonough strain (fms)-like tyrosine kinase receptor Flt-1 (VEGFR1) and the fetal liver kinase-1 receptor Flk-1 (VEGFR2), also called KDR [3]. Expression of these receptors increases under pathological conditions in which hypoxia is a main feature [4]. VEGF binds to its receptors and stimulates a variety of signaling molecules, resulting in promotion of neovascularization [5-7]. Both extracellular signal-regulated kinase (ERK) and protein kinase C (PKC) are activated by VEGF and contribute to the induction of endothelial cell proliferation and migration that are essential for regulation of angiogenesis [8,9].

Annexin A2, a cytosolic phospholipid and Ca^2+^ binding protein, is a receptor of many angiogenesis-related proteins [10], such as angiostatin and tissue plasminogen activator (t-PA). Annexin A2 can form heterotetrameric complexes on the surface of endothelial cells with the annexin A2 light chain (called S100A10 or p11), and this stimulates generation of t-PA dependent plasmin [11]. Plasmin is a highly reactive enzyme that is physiologically involved in fibrinolysis and plays an important role in neoangiogenesis [12]. In addition, annexin A2 is a substrate of PKCα, PKCβI, and PKCβII kinases in cells. Phosphorylation of annexin A2 serine 25 is associated with its nuclear entry, DNA synthesis, and cell proliferation [13]. However, annexin A2 has not been reported to participate in other angiogenetic mechanisms, such as the VEGF/VEGFR1 or VEGF/VEGFR2 pathways in pathological neovascularization.

The function and regulatory role of annexin A2 in retinal neovascularization have not been studied extensively. Here we describe a preliminary investigation of the expression of annexin A2, its effect on angiogenesis, and its functional relationship with VEGF in a mouse model of ischemia-induced retinal neovascularization model and in RF/6A cells.

## Methods

The following materials were used. Recombinant VEGF and recombinant VEGFR2 were from Strathmann Biotech (Hanover, Germany). Interferon-α (TNF-α), Interleukin 1-β (IL1-β), fibroblast growth factor-2 (FGF2), placenta growth factor (PIGF), anti–VEGF monkey mAb, and anti–VEGFR2 monkey mAb were from R&D Systems (Minneapolis, MN); calphostin C, LY333531, rottlerin, SU10944, GF1092023, U0126, and PD98059 were from Biomol International (Plymouth Meeting, PA). Actinomycin D was from CalBiochem (San Diego, CA), and complete mini–proteinase inhibitor cocktail tablets were from Roche Diagnostic (Mannheim, Germany). Other chemicals and reagents were obtained from Sigma Chemical Co (St. Louis, MO). unless otherwise indicated.

### Construction of adenoviral vector expressing mouse annexin A2

Production of adenoviral vectors that express mouse annexin A2 (Ad annexin A2) has been described previously [14]. Briefly, full-length mouse Annexin A2 cDNA was amplified by PCR with the primer set of 5′-GAG GAT CCA TGT CTA CTG TTC ACG AA-3′ and 5′-GGA CTA GTT CAT CTC CAC CAC ACA-3′. After double digestion with BamH I and Spe I, human annexin A2, was cloned into pENTR/CMV-EGFP vector through corresponding sites. The expression of the EGFP-Annexin A2 fusion protein was verified by western blot and fluorescence microscopy. An adenovirus vector expression Kit (TaKaRa, Ohtsu, Japan) was used to achieve in vivo homologous recombination between the transfer cassette bearing the annexin A2 expression unit and the adenovirus genome, as well as the restriction enzyme-digested adenovirus genome tagged with terminal protein in 293 cells. Plaque-purified adenoviruses were propagated in 293 cells. The viral lysates were purified and concentrated through double rounds of CsCl step gradients.

### Cell culture

The choroid-retinal endothelial cells line RF/6Awas obtained from the cell bank of the Chinese Academy of Science in Shanghai, China. RF/6A cells were cultured in Ham's-F12K medium (Gibco Life Technologies, Eggenstein, Germany), supplemented with 5% fetal calf serum, 100 U/ml penicillin, and 0.1 mg/ml streptomycin at 37 °C in a humidified atmosphere of normal air. Fresh normoxic or hypoxic (degassed) medium containing 1% fetal bovine serum (FBS) was added to the cells, and the cultures were placed in a Therma Forma Series II water-jacketed CO_2_ incubator (Marietta, OH) and perfused with 1% O_2,_ 94% N_2_, 5% CO_2_ or incubated under normoxic conditions at 37 °C for 1, 3, 6, 12, or 24 h. For VEGF and annexin A2 activity studies, the RF/6A cells were subjected to hypoxic conditions for 2 or 4 h followed by incubation under normoxic conditions for an additional 12 h [15].

### Mouse model of hypoxia-induced ischemic retinopathy

The model of hypoxia-induced retinopathy has been described previously [16]. C57BL/6J mice were bought from the Experimental Animal Center of Second Military Medical University, Shanghai, China. We used a hypoxia-induced retinopathy in which mouse pups with their nursing mothers were exposed to 75% O_2_ from postnatal day 7 (P7) to P12. On P12, mice were returned to room air for 5 days [17]. The animals were maintained in standard shoebox cages with water and food at a constant temperature of 23±1 °C and on a 12-h light–dark cycle. Oxygen concentration was measured with an oximeter (Toptronic, Milan, Italy). At the end of the oxygen exposure (day 12) and 5 days after return to normoxic conditions, the pups were killed, and retinal alterations were observed. Mice of the same strain and of the same age were kept in room air and used as control subjects. At various time points, mice received intravitreous injections of siRNA or adenoviral vector, as will be described in the paragraphs that follow. The study protocol adhered to the ARVO Statement for the Use of Animals in Ophthalmic and Vision Research.

### Analysis of annexin A2 mRNA half-life

To determine whether the increase in annexin A2 mRNA was caused by an increase in transcription, RF/6A cells were exposed to 5 µg/ml actinomycin D after 1 h of incubation with vehicle or 10 ng VEGF. The total RNA was then extracted, and real-time PCR analysis was performed.

### Real-time PCR analysis

Total RNA was isolated from retinas and cells using an RNeasy kit (TRIzol; Invitrogen, Carlsbad, CA). Single-stranded cDNA was synthesized from 1 µg total RNA using an oligo (dT) 18-mer as primer, and reverse transcription (MMLV Reverse Transcriptase; Invitrogen) in a final reaction volume of 25 µl. Real-time PCR was performed (QuantiTect SYBR Green PCR Kit; Qiagen, Valencia, CA) with a light-cycling system (LightCycler; Roche Diagnostics GmbH, Mannheim, Germany). Primers were described in Table 1. The value of annexin A2 mRNA expression were normalized to GAPDH gene expression.

**Table 1:** Primers used for RT–PCR analysis

**Gene**	**Primers (3′-5′)**
*annexin A2*	F: CAATGCACAGAGGCAGGACAT
	R: TTGGTTCTTGAGCAGA TGATC
*monkey annexin A2*	F: AGCAGAGCACAGCCGTGCTA
	R: TGTGGACGAGTCATACTGCG
*mouse GAPDH*	F: CCCAGCATTCAAGAAACCATCT
	R: CGACCCTGAACCTTTGAGTG
*monkey GAPDH*	F: GTCGTGGAGTCTACTGGCGTC
	R: ATGA GCCCTTCCACGATGC

Primer sequences were designed with Primer ExpressTM 2.0 (Applied Biosystems) software. Probes were labeled with 6-carboxyfluorescein (FAM) and 6-carboxy-N,N,N´,N´- tetramethylrhodamine (TAMRA).

### Western blot analysis for annexin A2 and VEGF

Mouse eyeballs were lysed with lysis buffer that consisted of 10 mM HEPES, pH 7.9, 10 mM potassium chloride, 0.1 mM EDTA, 1 mM dithiothreitol, 1% NP-40, and protease inhibitor cocktail (Roche). The mice were anesthetized by intraperitoneal injection of Avertin (1.2% tribromoethanol and 2.4% amylene hydrate in distilled water, 0.02 ml/g bodyweight; Sigma-Aldrich, St. Louis, MO). Briefly, anesthetized mice were killed by cervical dislocation, and the eyes were rapidly removed. Next, 100 µg whole-cell extracts were resolved in 10% SDS-polyacrylamide gels and electrophoretically transferred onto a polyvinylidene difluoride membrane (Millipore, Bedford, MA). For detection of mouse annexin A2, rabbit anti-mouse monoclonal antibody was used. Detection was performed with HRP-conjugated goat anti-rabbit immunoglobulins (Biosource. International, Camarillo, CA) and enhanced chemiluminescence (Amersham Bioscience UK Ltd., Little Chalfont, UK). After analysis, goat anti-GAPDH antibody (I-19; Santa Cruz Biotechnology, Santa Cruz, CA) was used to verify equal protein loading and transfer.

### siRNA interference in RF/6A cells and in vivo

Small RNA of annexin A2 (Si annexin A2) was synthesized using a Silencer siRNA Construction Kit (Ambion, Austin, TX). The following DNA templates were used to synthesize double-stranded Si annexin A2: sense, 5′-CAA TGC ACA GAG GCA GGA CAT-3′ and anti-sense, 5′-TTG GTT CTT GAG CAG ATG ATC-3′. An unrelated oligonucleotide (Si annexin A2_M) recognizing an irrelevant transcript was used as a negative control. Transfection was performed with the siPORT lipid transfection reagent (Ambion) following the manufacturer’s instructions. Cells were grown subconfluently on six-well plates and transfected with 200 nM of siRNA. After 1 day of culture, cells were analyzed further as specifically indicated [18]. In repeated experiments, the experimental results not showed significant difference between 24 h and 48 h.

### Intraocular injections

C57BL/6 mice were placed in 75% oxygen at P7, and at P12, mice (n=18) were returned to room air and given an intravitreous injection of 1 μg of Si annexin A2, Si annexin A2_M, Ad annexin A2, or Ad annexin A2_N [19]. For siRNA injections, RNA strands were annealed for 2 min at 94 °C in annealing buffer and siRNA was diluted in phosphate-buffered saline (PBS; 100 nM). Next, 1 μl containing 1 μg of SiRNA was injected. For vectors, 1 μl containing 10^10^ particle units (pu) was injected. Injections were done with a Harvard pump microinjection apparatus and pulled glass micropipettes. Each micropipette was calibrated to deliver 1 μl of vehicle upon depression of a foot switch. Mice were anesthetized with 10% chloraldurat (3.5ml/kg) given intraperitoneally, and their pupils were dilated with 1% tropicamide eye drops. Under a dissecting microscope, the sharpened tip of the micropipette was passed through the mouse sclera just behind the limbus into the vitreous cavity, and the foot switch was depressed.

### Immunofluorescent staining for annexin A2

At P17, mice were euthanized and eyes were removed and fixed for 30 min in 0.1 M phosphate buffer, pH 7.6, containing 5% paraformaldehyde and 5% sucrose. After 30 min, corneas and lenses were removed and then fixation was continued for another hour. After washing overnight with 0.1 M phosphate buffer containing 20% sucrose, the eyecups were frozen in optimum cutting temperature embedding compound (Miles Diagnostics, Elkhart, IN). Ocular frozen sections (10 µm) were dried with cold air for 20 min, fixed in freshly prepared 4% paraformaldehyde in 0.05 M PBS at room temperature for 15 min, and rinsed with 0.05 M Tris-buffered saline (TBS) for 10 min. Next, 10 μm frozen sections were fixed in 5% paraformaldehyde, and nonspecific binding was blocked by a 30 min incubation at room temperature in 10% normal goat serum. The sections were incubated for 1 h at room temperature with a 1:200 rabbit anti-mouse monoclonal antibody against annexin A2. Slides were washed for 3×2 min and incubated in 1:200 fluorescein isothiocyanate (FITC) -conjugated goat anti-rabbit (Invitrogen) for 30 min at room temperature. After washing with PBS for 3x2 min, slides were viewed with a Nikon fluorescence microscope.

At P15, mice reared in room air and mice with hypoxia-induced ischemic retinopathy were euthanized. Their eyes were rapidly removed and frozen in in an optimal cutting temperature compound. Sodium sections (10 μm) were cut. Frozen ocular sections were fixed with 5% paraformaldehyde for 15 min, washed with PBS three times for 5 min, and then immersed in chilled 50% methanol or 50% acetone for 15 min. The sections were washed with PBS and blocked with 10% normal goat serum for 30 min. A 1:150 dilution of rat anti-CD31 (anti-PECAM; BD PharMingen, San Diego, CA) and a 1:200 dilution of rabbit anti-mouse monoclonal antibody against annexin A2 were added, and the slides were incubated at 4 °C overnight. The sections were washed three times for 5 min, and incubated and incubated with a goat anti-rabbit IgG conjugated with FITC and Cy3-conjugated donkey anti-rat antibody (Invitrogen). After mounting, the sections were viewed with a Nikon fluorescence microscope.

### Quantification of retinal neovascularization

The eyes of sacrificed P17 mice were enucleated and fixed for 1 h at 4 °C with 4% paraformaldehyde. The retinas were dissected, and stained with FITC conjugated with Griffonia Simplicifolia Isolectin B4 (Sigma) for 12 h at 4 °C to detect vascular endothelial cells. The retinas were washed with PBS for 120 min (6×20 min). Four radial cuts were made in each retina, and retinas were mounted in mounting media. Retina pictures were taken by fluorescence microscopy and the integrity of the retina image was restored with Adobe^®^ Photoshop^®^ Version 7.0 (Adobe Systems, San Jose, CA). Neovascularization was quantified by comparing the number of pixels in the affected areas with the total number of pixels in the retina.

### Statistical analysis

All experiments were performed at least twice. Results are reported as mean±standard deviation. An unpaired Student’s *t*-test was used to determine statistical significance. A p<0.05 was considered significant.

## Results

### Effects of different growth factors on annexin A2 in vivo and in RF/6A cells

**Figure 1:**
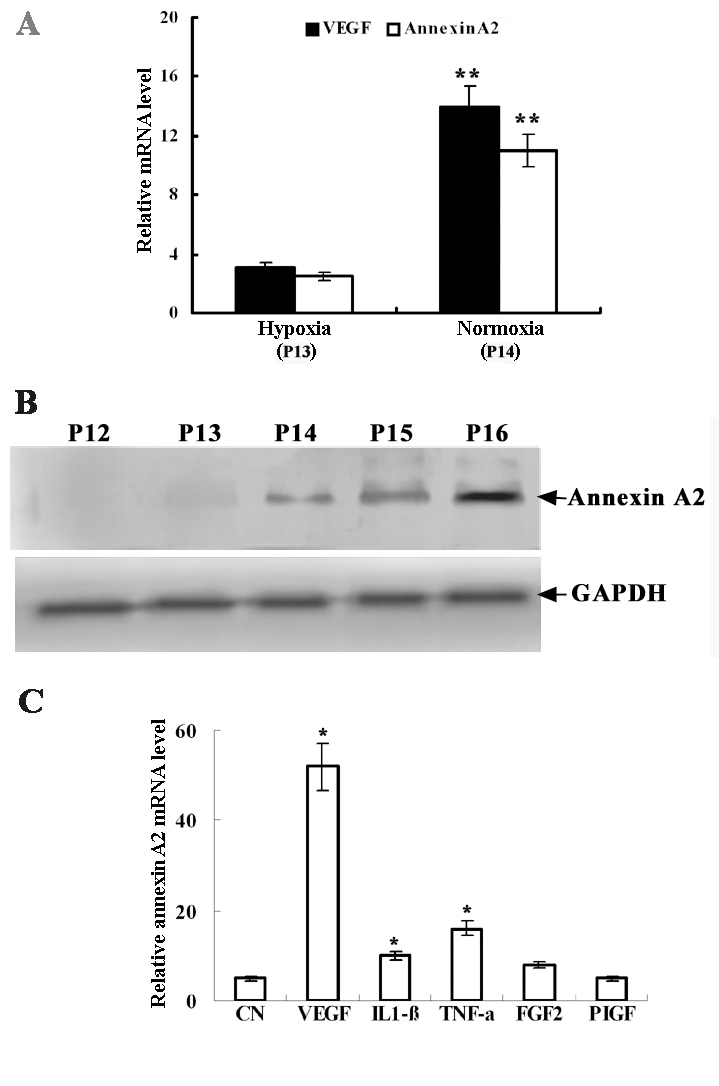
Annexin A2 expression levels increased in a mouse model of hypoxia-induced retinopathy and in vitro. Relative mRNA levels of Annexin A2 were normalized by GAPDH mRNA levels **A:** Levels of annexin A2 and VEGF mRNA were measured by quantitative real-time PCR in mouse retinas immediately after hypoxia treatment (P13) and 12 h after return to room air (P14). Double asterisks (**) indicate a p<0.01 compared with controls. Six mice were used for each time point. **B:** The figures showed western blot results for annexin A2 protein in retinas from the mouse model of hypoxia-induced retinopathy (days P12–P16). **C:** The figures showed the effects of cytokines factors on the expression of annexin A2 mRNA. RF/6A cells were treated respectively with VEGF, IL1-β, TNF-α, FGF2, or PIGF at 25 ng/ml for 2 h. CN was Annexin A2 expression levels in RF/6A cells untreated with the cytokines. Total RNA was isolated, and quantitative real-time PCR was used to detect the expression of annexin A2 mRNA. Results are representative of three independent experiments, each performed with duplicate samples. Asterisk (*) indicates a p<0.05 compared with untreated control.

Annexin A2 mRNA expression was investigated in the mouse model of hypoxia-induced retinopathy. As shown in Figure 1A, VEGF mRNA levels were significantly higher in P14 mice than in P13 mice (p<0.01). Annexin A2 mRNA levels were also significantly increased. mRNA levels in P14 mice were approximately four times the levels in P13 mice. Western blot analysis demonstrated that annexin A2 protein levels were elevated in retinas from P14 to P16 compared with P13 (Figure 1B). Several cytokines associated with angiogenesis were detected. Annexin A2 mRNA expression in RF/6A cells was dramatically upregulated by VEGF and modestly upregulated by TNF-α and IL-1β (Figure 1C). FGF2 and PIGF did not significantly affect the expression of annexin A2 mRNA.

### Effect of VEGF on annexin A2 and half-life of annexin A2 mRNA in RF/6As

**Figure 2:**
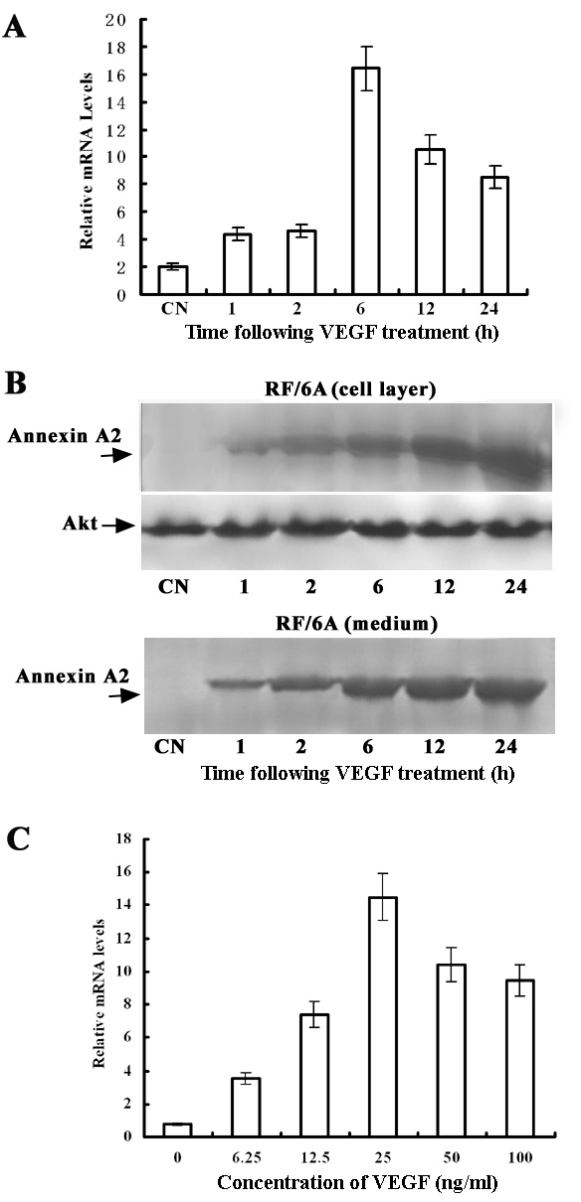
VEGF induces annexin A2 mRNA and protein expression in a time-dependent and dosage-dependent manner in RF/6A cells. **A:** RF/6A cells were treated with 25 ng/ml VEGF for the indicated length of time. Results of quantitative real-time PCR are representative of three independent experiments, each performed with duplicate samples. **B**: The protein expression of Annexin A2 was analyzed by western blot in RF/6A cells treated with 25 ng/ml. VEGF for the indicated length of time. Total cell lysates and acetone-precipitated medium from cells were analyzed for the presence of annexin A2 protein. Annexin A2 is indicated (arrows). The same membrane was blotted with anti–total Akt antibody for normalization. **C:** VEGF induced an increase in annexin A2 mRNA levels measured by quantitative real-time PCR in a concentration-dependent manner. RF/6A cells were treated with the indicated concentrations of VEGF for 2 h, and then total RNA was isolated.

Annexin A2 expression was stimulated after treatment with 25 ng/ml VEGF. Annexin A2 mRNA expression peaked at 6 h and persisted for 24 h after treatment (Figure 2A). Western blot analysis showed similar results in the cell layer and medium (Figure 2B). VEGF upregulated annexin A2 expression in a dose-dependent manner, with a peak effect at 25 ng/ml (Figure 2C).

**Figure 3:**
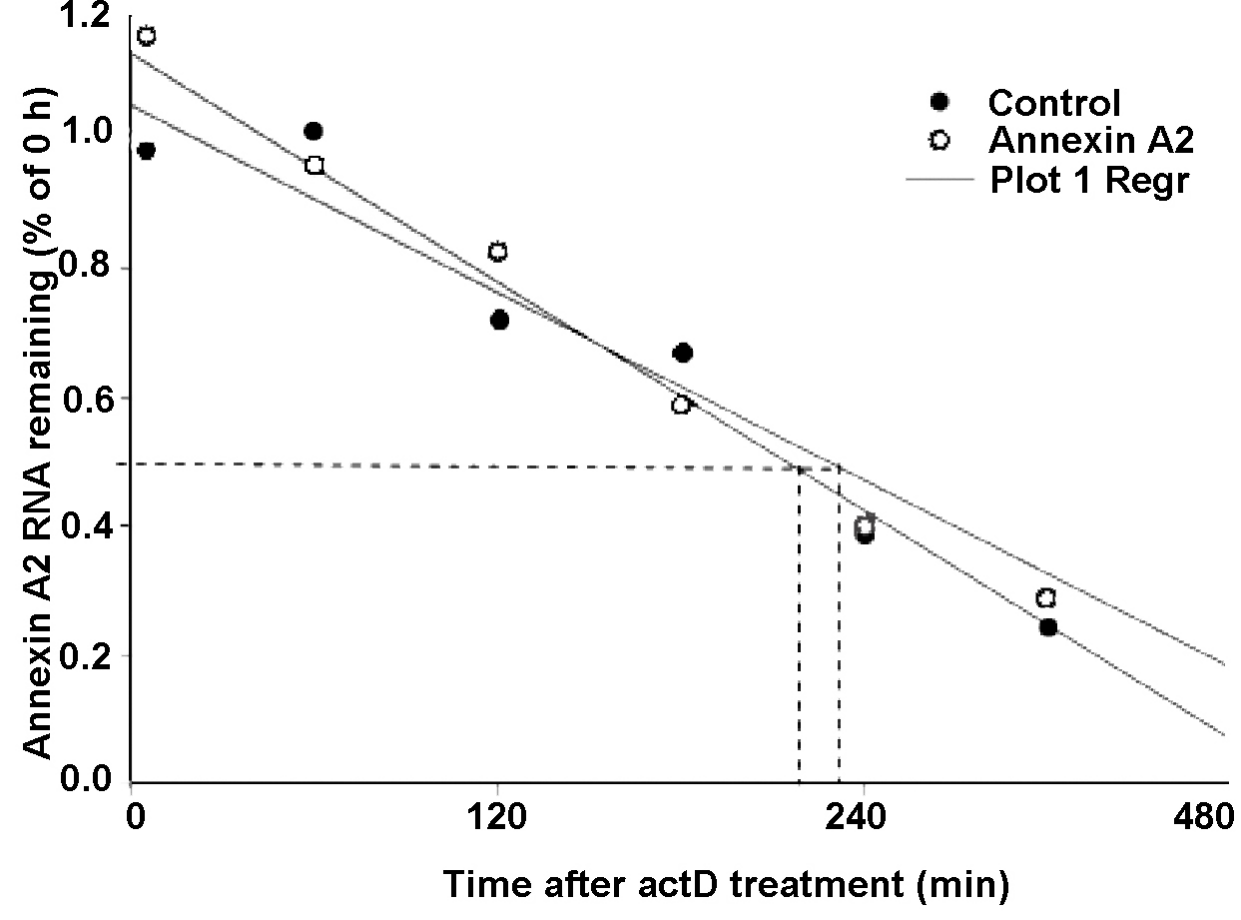
Effects of VEGF on annexin A2 mRNA half-life. RF/6A cells were treated with 25 ng/ml VEGF for 1 h. Half the plates were returned to OptiMEM plus 0.5% FBS without VEGF, and 5 µg/ml actinomycin D (actD) was added to all plates. Total RNA was isolated at the indicated time points after actD treatment, and real-time PCR was performed. Representative data from three independent experiments are shown.

To investigate whether VEGF affected the stability of annexin A2 mRNA, we measured the half-life of annexin A2 mRNA in the presence of actinomycin D, which inhibits de novo gene transcription. As shown in Figure 3, the half-life of annexin A2 mRNA was 215 min in unstimulated cells and 230 min in cells treated with VEGF. There was no significant difference between control and VEGF treatment, suggesting that the increase in annexin A2 mRNA levels in response to VEGF treatment was caused by transcriptional activation.

### Effects of VEGFR2 on VEGF-stimulated annexin A2 upregulation

**Figure 4:**
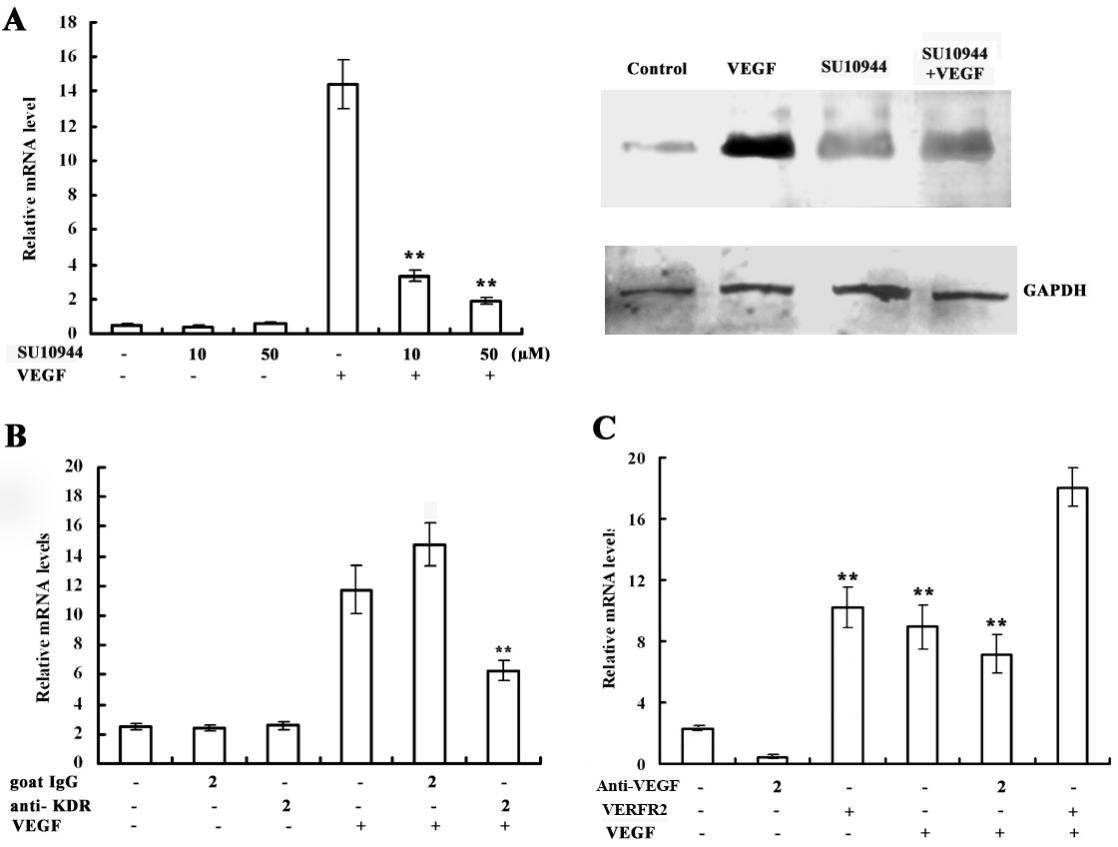
VEGF increases annexin A2 expression through VEGFR2. **A:** RF/6A cells were pretreated with 10 µM or 100 µM SU10944 for 30 min and then treated with 25 ng/ml of VEGF for 1 h. Annexin A2 expression was detected by real-time PCR and western blot analysis. **B:** RF/6A cells were pretreated with 2 µg/ml goat IgG or neutralizing anti–VEGFR2 antibody for 1 h and then stimulated with 25 ng/ml of VEGF for 1 h. Total RNA was isolated, and quantitative real-time PCR was performed. **C:** RF/6A cells were pretreated with anti-VEGF antibody or VEGFR2 for 1 h, and then treated with 25 ng/ml VEGF for1 h. Annexin A2 expression was detected with real-time PCR. Double asterisks (**) represent p<0.01 compared with VEGF-treated and VEGFR2-treated samples.

The VEGF-induced expression levels of the mRNA and protein of annexin A2 respectively were suppressed by 87% and 75% in RF/6A cells after pretreatment with SU10944 (Figure 4A). VEGF-induced annexin A2 expression after pretreatment of RF/6A cells with 2 µg/ml neutralizing antibody against monkey VEGFR2 was suppressed by 69% (Figure 4B). The results indicate that VEGFR2 plays a major role in VEGF-induced annexin A2 expression, although we cannot exclude a role for VEGFR1. Pretreatment of RF/6A cells with 2 µg/ml antibody against monkey VEGF almost entirely inhibited ischemia-induced annexin A2 expression (Figure 4C). The expression levels of annexin A2 rose markedly in the simultaneous presence of VEGF and VEGFR2 compared with VEGF or VEGFR2 singly (p<0.001).

### Different roles of protein kinase C (PKC) and mitogen activated protein kinase kinase (MEK) pathways

**Figure 5:**
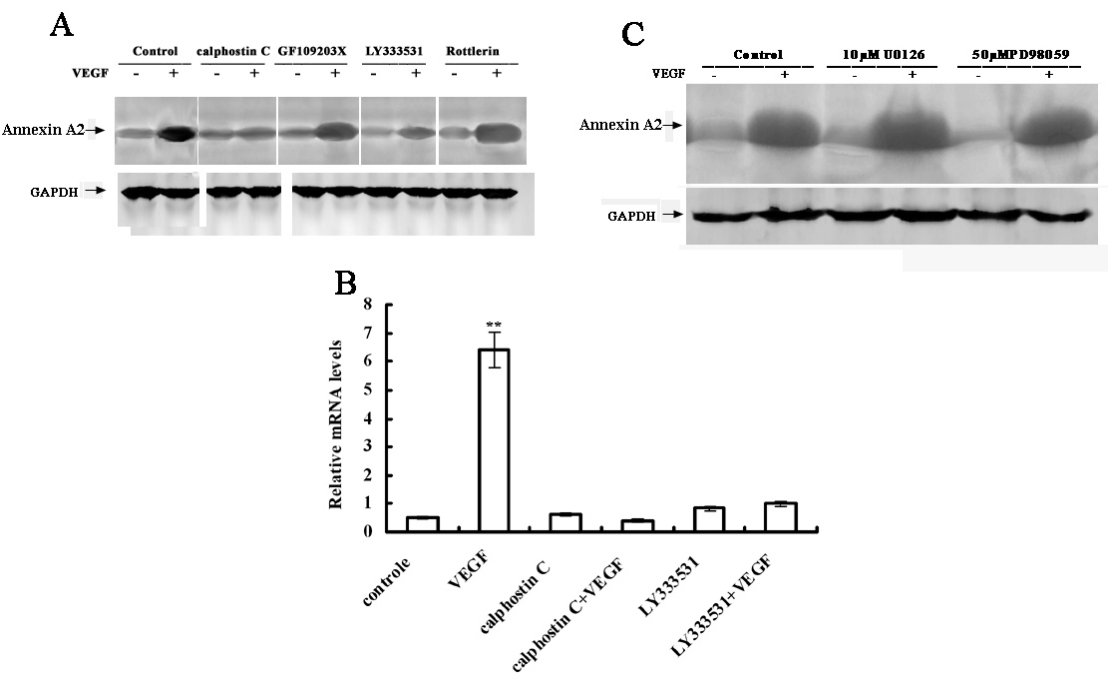
VEGF-induced annexin A2 expression was PKC-dependent. **A:** Serum-starved RF/6A cells were pretreated with 1 µM calphostin C, 3 µM GF109203X, 3 µM LY333531, or 5 µM rottlerin for 30 min and then treated with 25 ng/ml VEGF for 6 h. Annexin A2 protein was detected by western blotting. **B:** After overnight serum starvation, RF/6A cells were pretreated with 1 µM calphostin C or 2 µM LY333531 for 30 min and then were treated with 25 ng/ml VEGF for 2 h. Total RNA was isolated, and quantitative real-time PCR was used to detect the expression of annexin A2 mRNA. Double asterisk (**) indicates p<0.01 compared with control samples. **C:** Serum-starved RF/6A cells were pretreated with 10 µM U0126 or 50 µM PD98059 for 30 min and then were treated with 25 ng/ml VEGF for 6 h. Total cell lysates were subjected to western blot analysis for annexin A2. The same membrane was blotted with anti–GAPDH antibody for normalization.

The role of the PKC pathway was investigated in VEGF-induced annexin A2 upregulation because PKC is a well documented downstream target of VEGF. RF/6A cells were pretreated with PKC inhibitors. As shown in Figure 5A,B, calphostin C and LY333531 of PKC inhibitors inhibited VEGF-induced upregulation of annexin A2. Inhibition of PKCβ with LY333531 reduced annexin A2 protein expression by 80%, whereas inhibition of PKC with rottlerin resulted in only slight inhibition, indicating that a PKC isoform is involved in VEGF-induced annexin A2 upregulation. The Mitogen-activated protein (MAP)/ERK kinase (MEK) pathway was also investigated; two different inhibitors of MEK (U0126 or PD98059) failed to block VEGF-induced annexin A2 upregulation (Figure 5C). Taken together, these results demonstrate that VEGF-induced annexin A2 expression was related to PKC signaling.

### Annexin A2 promotes retinal neovascularization in ischemic retina

Annexin A2 has been shown to promote neovascularization in tumors [20]. To determine the effect of annexin A2 on retinal neovascularization in vivo, we employed two strategies: 1) express annexin A2 in the retinas of mice with hypoxia-induced ischemic retinopathy by intraocular injection of Ad annexin A2; and 2) intraocular injection of Si annexin A2.

**Figure 6:**
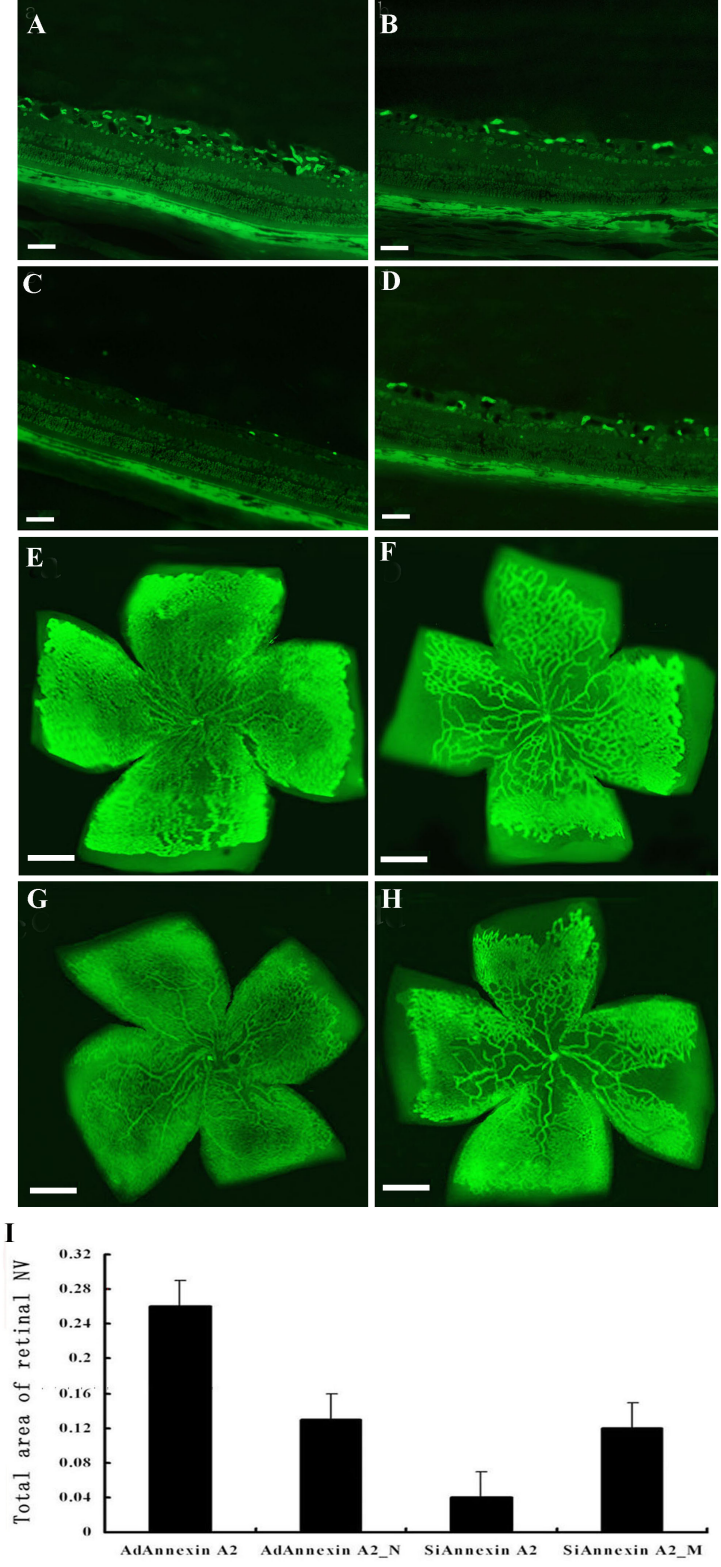
Annexin A2 promotes retinal neovascularization in mice with ischemic retinopathy. Mice at P7 were reared in 75% oxygen for five days followed by room air for five days. Eyes of mice (n=12) were injected with (**A**) Ad annexin A2, (**B**) Ad annexin A2_N, (**C**) Si annexin A2 or (**D**) Ad annexin A2_N at P12. Retinas were isolated and stained with (**A-D**, scale bar represents 100 μm) FITC-antibody for annexin A2 (n=6) to detect expression of annexin A2, or (**G-H**) isolectin B4 (n=6) to detect neovascularization at P17. Magnification is 5X. The scale bar represents 400 μm. **I:** Measurement of the area of neovascularization in the retina by image analysis (mm^2^) showed that eyes injected with SiAnnexin A2 had significantly less neovascularization than eyes injected with AdAnnexin A2.

Mice with ischemic retinopathy who received intravitreous injections of Ad annexin A2, Ad annexin A2_N, Si annexin A2, or Si annexin A2_M at P12 were euthanized at P17. Immunofluorescent staining using an antibody that specifically recognizes mouse annexin A2 showed staining along the surface of the retina in eyes injected with Ad annexin A2 (Figure 6A), Ad annexin A2_N (Figure 6B), and Si annexin A2_M (Figure 6D), but the highest expression levels of annexin A2 were in Ad annexin A2-treated mice. Immunofluorescent staining did not show any annexin A2 in the retinas of Si annexin A2-treated mice (Figure 6C).

Representative pictures of isolectin B4-stained retinal vasculature in mice at P17 are shown in Figure 6E-H. Ad annexin A2-treated mice showed a marked increase in multiple neovascular tufts, showing neovascularization throughout the retina (Figure 6E). In contrast, Si annexin A2-treated mice (Figure 6G) showed an apparent decrease in neovascularization compared with Ad annexin A2_N (Figure 6F) and Si annexin A2_M-treated mice (Figure 6H).

Measurement of the areas of retinal neovascularization showed that there was significantly more neovascularization in eyes injected with Ad annexin A2 than in eyes injected with Ad annexin A2_N. Furthermore, Si annexin A2-treated mice had significantly smaller areas of neovascularization in their retinas than Si annexin A2_M-treated mice. The neovascularization area did not differ between the two groups treated with Ad annexin A2_N and Si annexin A2_M (Figure 6I).

### Endogenous annexin A2 was increased in endothelial cells in ischemic retina

**Figure 7:**
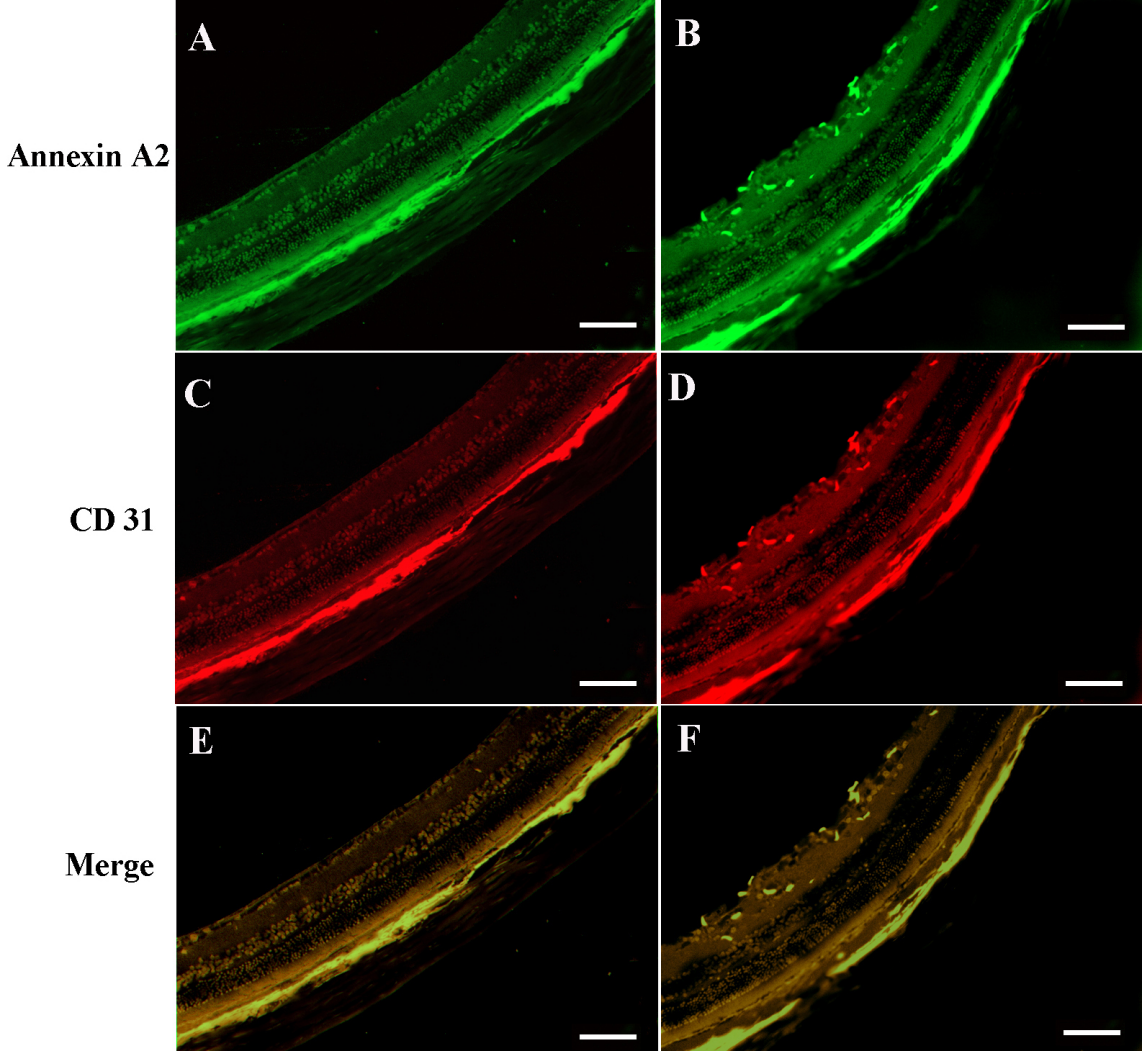
The annexin A2 expression in vascular endothelial cells . At P15, mice reared in room air (**A, E, C**) or mice with hypoxia-induced retinopathy (**B, D, F**) were killed (n=6), and ocular frozen sections were stained with a rat antibody directed against CD31 or a rabbit anti-murine annexin A2 antibody (**B, D, F**). Secondary antibodies were a goat anti-rabbit IgG conjugated with FITC and a Cy3-conjugated donkey anti-rat antibody. Small cross-sections of retinal vessels are evident in the CD31-stained retina (red) from a normal mouse, while dilated vessels and new vessels can be seen in the CD31-stained retina from a mouse with ischemic retinopathy. There is no detectable staining for annexin A2 in the retina of the mouse reared in room air, but there is prominent staining in the retina of the mouse with ischemic retinopathy (green). Merging of the images for the retina of the mouse with ischemic retinopathy (bottom, right) by simultaneously viewing with the red and green channels, demonstrates colocalization of CD31 and annexin A2, indicating that annexin A2 is expressed in vascular endothelial cells. Scale bar represents 100 μm.

A polyclonal rabbit anti-murine annexin A2 antibody was used to localize endogenous annexin A2 in mouse retinas. At P15, mice that had been reared in room air had normal retinas and no detectable staining for annexin A2 (Figure 7A), yet retinopathy showed prominent staining that colocalized with CD31, which selectively stains endothelial cells (Figure 7D). This indicates that annexin A2 was upregulated in vascular endothelial cells in ischemic retina neovascularization.

## Discussion

In this study, we have demonstrated that annexin A2 expression is increased in the retina under hypoxic conditions. This expression of annexin A2 is regulated by many functional proteins, such as VEGF, VEGFR2, and PKC. Suppressing the ischemia-induced expression of VEGFR2 with anti–VEGFR2 monoclonal antibody, or of PKC with PKC inhibitors (Calphostin C, LY333531) suppressed the increase in annexin A2 mRNA. This suggests that VEGF, VEGFR2, and PKC may be involved in regulating the expression of annexin A2, and that VEGF and VEGFR2 may play major roles.

These studies in vitro show that the annexin A2 expression of the mRNA and protein in RF/6A cells are dramatically upregulated by VEGF (Figure 1C and Figure 2) in a dose-dependent manner, and the increase in annexin A2 levels of the mRNA and protein in response to VEGF treatment was caused by transcriptional activation (Figure 3). These indicate that annexin A2 is upregulated by VEGF in vascular endothelial cells in ischemic retina.

Immunofluorescent staining with an antibody that recognizes murine annexin A2 did not show a detectable signal in normal retinas, but ischemic retinas of age-matched mice showed staining for annexin A2 on the retina surface and in vascular endothelial cells. This upregulation of endogenous annexin A2 may not be a major factor like VEGF in encouraging retinal neovascularization, but it modulates the response; knockdown of annexin A2 by injection of Si annexin A2 significantly decreased neovascularization compared with control eyes injected with Si annexin A2_M. Furthermore, bolstering endogenous levels of annexin A2 by injecting an Ad annexin A2 vector significantly increased retinal neovascularization. Therefore, annexin A2 may act in a positive feedback loop to help promote neovascularization in the retina, as has been postulated for tumors [21,22].

Retinal neovascularization is a prevalent cause of blindness and the focus of an intense effort to find selective molecular treatments. This effort has received an important boost from demonstrations that VEGF is a stimulator for both retinal and choroidal neovascularization [23]. Retinal ischemia (or hypoxia) is the central pathogenic feature of retinal neovascularization and one of its major consequences is upregulation of VEGF [24]. Retinal neovascularization is suppressed by agents that bind VEGF [25,26] or block VEGF receptors [27,28]. Although retinal ischemia (or hypoxia) is the central pathogenic feature of retinal neovascularization, VEGF is a necessary stimulator, because VEGF receptor kinase inhibitors or other agents that bind VEGF strongly suppress retinal neovascularization [29-31]. Even though the mechanism of the synergistic effect of annexin A2 is unknown in ischemia retina, there is also no evidence appearing to directly effect of annexin A2 on VEGF; it may have synergistic activity in combination with agents that bind VEGF or by binding the mRNA of VEGF.

**Figure 8:**
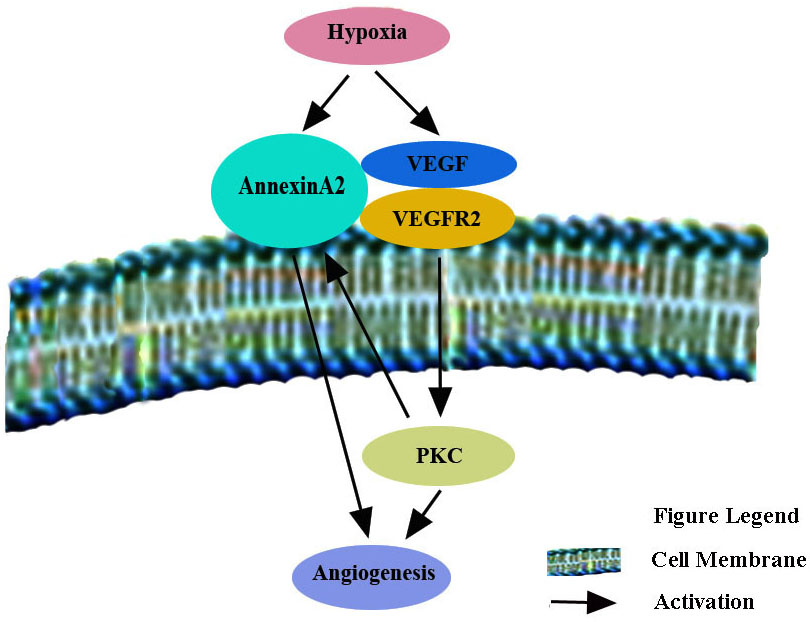
Diagram of signal transduction cascades investigated in this study. In hypoxia, VEGF and annexin A2 transcription was upregulated by hypoxia inducible factor. VEGF directly effect on the expression of annexin A2, or a combination of VEGF and VEGFR2 induced the expression of annexin A2 on the surface of the cell membrane. VEGF and VEGFR2 also may be promote Annexin A2 expression by PKC pathway. Annexin A2 further influenced neovascularization.

These data support the hypothesis that annexin A2 acts as an activator of retinal neovascularization that is upregulated by VEGFR2 or a conjugate of VEGF and VEGFR2 (Figure 8). This is well illustrated by the relationship between annexin A2 and VEGF (Figure 8). In hypoxia, VEGF and annexin A2 transcription are upregulated by hypoxia inducible factor [32-34]. The biologic activity of secreted VEGF is further influenced by hypoxia-inducible expression of VEGFR2 [35-37]. At the same time, VEGF directly effect on the expression of annexin A2, or a combination of VEGF and VEGFR2 induces the expression of annexin A2. Annexin A2 is a substrate protein of PKC. Activation of annexin A2 phosphorylation is promoted by the PKC signaling pathway which promote ischemic retina neovascularization. Otherwise, serine 25 phosphorylation of annexin A2 may be associated with the nuclear entry of annexin A2, mRNA expression of VEGF, and endothelial cell proliferation. Therefore, on one hand, annexin A2 as one of the receptors for plasminogen and tPA is involved in the angiogenic process [38]. On the other hand, presumably annexin A2 as a mRNA binding protein influences the stability of mRNA expression of VEGF, or is involved in the angiogenic process by VEGF/VEGFR pathway.

In summary, we have demonstrated that increased expression of annexin A2 in ischemic retinas is induced by increased levels of VEGF mRNA. This inductive effect of VEGF for annexin A2 is mainly regulated by the expression level of VEGFR2 mRNA. Blocking the ischemia-induced rise in annexin A2 mRNA with Si annexin A2 also inhibits retinal neovascularization in ischemic retinas. Together our data clearly identify annexin A2 as a new functional protein in VEGF expression in retinal neovascularization induced by hypoxia. Compelling evidence from the literature and our own findings suggest that annexin A2 may be a potential target for the development of effective therapeutic strategies for the treatment of retinal neovascularization.

## References

[1] Iranmanesh R, Eandi CM, Peiretti E, Klais CM, Garuti S, Goldberg DE, Slakter JS, Yannuzzi LA (2007). The nature and frequency of neovascular age-related macular degeneration.. Eur J Ophthalmol.

[2] Das A, McGuire PG (2003). Retinal and choroidal angiogenesis: pathophysiology and strategies for inhibition.. Prog Retin Eye Res.

[3] Ambati J, Ambati BK, Yoo SH, Ianchulev S, Adamis AP (2003). Age-related macular degeneration: etiology, pathogenesis, and therapeutic strategies.. Surv Ophthalmol.

[4] Hanahan D, Folkman J (1996). Patterns and emerging mechanisms of the angiogenic switch during tumorigenesis.. Cell.

[5] Ferrara N, Gerber HP, LeCouter J (2003). The biology of VEGF and its receptors.. Nat Med.

[6] Boccellino M, Giovane A, Servillo L, Balestrieri C, Quagliuolo L (2002). Fatty acid mobilized by the vascular endothelial growth factor in human endothelial cells.. Lipids.

[7] Gerke V, Moss SE (2002). Annexins: from structure to function.. Physiol Rev.

[8] Rescher U, Gerke V (2004). Annexins–unique membrane binding proteins with diverse functions.. J Cell Sci.

[9] Ferrara N (2004). Vascular Endothelial Growth Factor: Basic Science and Clinical Progress.. Endocr Rev.

[10] Cross MJ, Dixelius J, Matsumoto T, Claesson-Welsh L (2003). VEGF-receptor signal transduction.. Trends Biochem Sci.

[11] Sharma MC, Sharma M (2007). The role of annexin II in angiogenesis and tumor progression: a potential therapeutic target.. Curr Pharm Des.

[12] Sharma MR, Koltowski L, Ownbey RT, Tuszynski GP, Sharma MC (2006). Angiogenesis-associated protein annexin II in breast cancer: selective expression in invasive breast cancer and contribution to tumor invasion and progression.. Exp Mol Pathol.

[13] Syed SP, Martin AM, Haupt HM, Arenas-Elliot CP, Brooks JJ (2007). Angiostatin receptor annexin II in vascular tumors including angiosarcoma.. Hum Pathol.

[14] Ottino P, Finley J, Rojo E, Ottlecz A, Lambrou GN, Bazan HEP, Bazan NG (2004). Hypoxia activates matrix metalloproteinase expression and the VEGF system in monkey choroid-retinal endothelial cells: Involvement of cytosolic phospholipase A2 activity.. Mol Vis.

[15] Smith LE, Wesolowski E, McLellan A, Kostyk SK, D'Amato R, Sullivan R, D'Amore PA (1994). Oxygen-induced retinopathy in the mouse.. Invest Ophthalmol Vis Sci.

[16] D'Amore PA (1994). Mechanisms of retinal and choroidal neovascularization.. Invest Ophthalmol Vis Sci.

[17] Aiello LP (1997). Clinical implications of vascular growth factors in proliferative retinopathies.. Curr Opin Ophthalmol.

[18] Tong JP, Yao YF (2006). Contribution of VEGF and PEDF to choroidal angiogenesis: a need for balanced expressions.. Clin Biochem.

[19] Ohno-Matsui K (2003). Molecular mechanism for choroidal neovascularization in age-related macular degeneration.. Nippon Ganka Gakkai Zasshi.

[20] Perez-Pinera P, Berenson JR, Deuel TF (2008). Pleiotrophin, a multifunctional angiogenic factor: mechanisms and pathways in normal and pathological angiogenesis.. Curr Opin Hematol.

[21] Folkman J (1995). Angiogenesis in cancer, vascular, rheumatoid and other disease.. Nat Med.

[22] Benedetta Donati M, Gozdzikiewicz J (2008). Angiogenesis and the progress of vascular and tumor biology: A tribute to Judah Folkman.. Thromb Haemost.

[23] Hayes MJ, Longbottom RE, Evans MA, Moss SE (2007). Annexinopathies.. Subcell Biochem.

[24] Moses MA (1997). The regulation of neovascularization of matrix metalloproteinases and their inhibitors.. Stem Cells.

[25] Berglin L, Sarman S, van der Ploeg I, Steen B, Ming Y, Itohara S, Seregard S, Kvanta A (2003). Reduced choroidal neovascular membrane formation in matrix metalloproteinase-2-deficient mice.. Invest Ophthalmol Vis Sci.

[26] Hwang J, Hodis HN, Hsiai TK, Asatryan L, Sevanian A (2006). Role of annexin II in estrogen-induced macrophage matrix metalloproteinase-9 activity: the modulating effect of statins.. Atherosclerosis.

[27] Nezi L, Greco D, Nitsch L, Garbi C (2002). The role of proteases in fibronectin matrix remodeling in thyroid epithelial cell monolayer cultures.. Biol Chem.

[28] Haas TL (2005). Endothelial cell regulation of matrix metalloproteinases.. Can J Physiol Pharmacol.

[29] Babbin BA, Parkos CA, Mandell KJ, Winfree LM, Laur O, Ivanov AI, Nusrat A (2007). Annexin 2 regulates intestinal epithelial cell spreading and wound closure through Rho-related signaling.. Am J Pathol.

[30] Garver WS, Hossain GS, Winscott MM, Heidenreich RA (1999). The Npc1 mutation causes an altered expression of caveolin-1, annexin II and protein kinases and phosphorylation of caveolin-1 and annexin II in murine livers.. Biochim Biophys Acta.

[31] Yan G, Luo W, Lu Z, Luo X, Li L, Liu S, Liu Y, Tang M, Dong Z, Cao Y (2007). Epstein-Barr virus latent membrane protein 1 mediates phosphorylation and nuclear translocation of annexin A2 by activating PKC pathway.. Cell Signal.

[32] Xia P, Aiello LP, Ishii H, Jiang ZY, Park DJ, Robinson GS, Takagi H, Newsome WP, Jirousek MR, King GL (1996). Characterization of vascular endothelial growth factor’s effect on the activation of protein kinase C, its isoforms, and endothelial cell growth.. J Clin Invest.

[33] Chiang Y, Rizzino A, Sibenaller ZA, Wold MS, Vishwanatha JK (1999). Specific down-regulation of annexin II expression in human cells interferes with cell proliferation.. Mol Cell Biochem.

[34] Wolberg AS, Roubey RA (2005). Annexin A2: better left alone.. Blood.

[35] Zhang J, McCrae KR (2005). Annexin A2 mediates endothelial cell activation by antiphospholipid/anti-beta2 glycoprotein I antibodies.. Blood.

[36] Saint-Geniez M, Maharaj AS, Walshe TE, Tucker BA, Sekiyama E, Kurihara T, Darland DC, Young MJ, D'Amore PA (2008). Endogenous VEGF is required for visual function: evidence for a survival role on müller cells and photoreceptors.. PLoS One.

[37] Stalmans I (2005). Role of the vascular endothelial growth factor isoforms in retinal angiogenesis and DiGeorge syndrome.. Verh K Acad Geneeskd Belg.

[38] Swisher JF, Khatri U, Feldman GM (2007). Annexin A2 is a soluble mediator of macrophage activation.. J Leukoc Biol.

